# Cervicovaginal natural antimicrobial expression in pregnancy and association with spontaneous preterm birth

**DOI:** 10.1038/s41598-020-68329-z

**Published:** 2020-07-21

**Authors:** Natasha L. Hezelgrave, Paul T. Seed, Evonne C. Chin-Smith, Alexandra E. Ridout, Andrew H. Shennan, Rachel M. Tribe

**Affiliations:** Department of Women and Children’s Health, School of Life Course Sciences, King’s College London, St Thomas’ Hospital, London, SE1 7EH UK

**Keywords:** Immunology, Innate immune cells, Biomarkers, Outcomes research, Infection, Inflammation

## Abstract

There is much interest in the role of innate immune system proteins (antimicrobial peptides) in the inflammatory process associated with spontaneous preterm birth (sPTB). After promising pilot work, we aimed to validate the association between the antimicrobial peptides/proteins elafin and cathelicidin and sPTB. An observational cohort study of 405 women at high-risk, and 214 women at low-risk of sPTB. Protein concentrations of elafin and cathelicidin, and the enzyme human neutrophil elastase (HNE) were measured in over 1,000 cervicovaginal fluid (CVF) samples (10 to 24 weeks’ gestation). Adjusted CVF cathelicidin and HNE concentrations (but not elafin) were raised in high-risk women who developed cervical shortening and who delivered prematurely and were predictive of sPTB < 37 weeks, with an area under the curve (AUC) of 0.75 (95% CI 0.68 to 0.81) for cathelicidin concentration at 14 to 15^+6^ weeks. Elafin concentrations were affected by gestation, body mass index and smoking. CVF elafin in early pregnancy was modestly predictive of sPTB < 34 weeks (AUC 0.63, 0.56–0.70). Alterations in innate immune response proteins in early pregnancy are predictive of sPTB. Further investigation is warranted to understand the drivers for this, and their potential to contribute towards clinically useful prediction techniques.

## Introduction

Spontaneous preterm birth (sPTB), defined as spontaneous birth prior to 37 weeks of gestation, is a leading cause of neonatal morbidity and mortality. Pathophysiologically, it may be initiated by a number of different and overlapping causative factors including intra-amniotic infection, decidual haemorrhage or senescence, cervical weakness, breakdown of maternal–fetal tolerance, declining progesterone activity^1^ and sterile inflammation^2^. The culmination of these pathological processes is a process of systemic inflammation, likely similar to the inflammatory pathway associated with early and established term labour (uterine and cervical neutrophil infiltration and release of cytokines, prostaglandins and matrix metalloproteins)^3–5^. Thus, increasing attention has been given to the potential use of inflammatory biomarkers to identify women at high risk of subsequent sPTB. These may have potential to guide prevention and/or management strategies, particularly if measurable in early pregnancy and linked to adverse outcome.

Natural antimicrobial peptides/proteins (NAPs), the body’s rapid ‘first defence’ response to foreign antigens, are small cationic host defence molecules functioning within the innate immune system. Expressed throughout the female genital tract at epithelial surfaces, their anti-protease action counteract the effect of protease mediated inflammation and tissue destruction^6^. NAPs exhibit antibacterial, antiviral and antifungal activity, largely attributed to disruption of microbial membranes^7^. Trappin2/elafin (also known as peptidase inhibitor 3) or skin-derived anti-proteinase (SKALP), secretory leucocyte protease inhibitor (SLPI), human β-defensins 1–3 and cathelicidin antimicrobial peptide (LL37/cathelicidin) are amongst the NAPs identified in the reproductive tract. Trappin2/elafin (referred to hereafter as elafin) expression has been reported to be raised in fetal membranes from women with preterm prelabour rupture of membranes (PPROM)^8^ and reduced in cervicovaginal fluid (CVF) from women with bacterial vaginosis (BV)^9^. Both cathelicidin and elafin have a relationship with human neutrophil elastase (HNE), a serine protease; cathelicidin is co-released with HNE from human neutrophils^10^ and HNE is inhibited by elafin^11^.

We previously reported in the ‘CLIC’ pilot study^12^ (n = 74 women at high risk of sPTB) that women who experienced cervical shortening (< 25 mm on transvaginal ultrasound scan prior to 24 weeks of gestation), a clinical indicator associated with sPTB, had raised concentrations of CVF elafin from 14 weeks of gestation (2.71 times higher, CI 1.94 to 3.79, p < 0.01) and that raised elafin concentration was associated with sPTB before 37 weeks. CVF cathelicidin concentration was also high around 18–19 weeks of gestation in those women who developed a short cervix.

On this basis, we hypothesised that CVF elafin and cathelicidin are potentially useful predictors of risk of cervical shortening and sPTB. The prospective observational cohort study ‘Insight’ was designed to validate these findings in an appropriately powered cohort. Firstly, the cohort was used to describe the longitudinal gestational profiles of CVF elafin, cathelicidin and HNE in women at high risk of sPTB, and low-risk controls, as well as their relationship with each other. Secondly, the relationships with cervical length, subsequent sPTB and adverse pregnancy outcomes were determined.

## Results

Of the 619 women with singleton pregnancies enrolled, 405 women were classified as high risk for sPTB and the remaining 214 as low risk for sPTB. The demographic characteristics of the cohort according to risk of sPTB at recruitment and birth outcome are shown in Tables 1 and 2 respectively. Primary outcome data (sPTB or term birth) was obtained for 611 out of 619 women. Within the group of high-risk women, 34% received a prophylactic or reactive intervention to prevent sPTB (cerclage, vaginal progesterone or Arabin pessary).

**Table 1:** Demographics of the INSIGHT cohort study population (all and by risk status).

Characteristic	Sub-category	High-risk women N = 405	Low-risk women N = 214	Comparison (difference/risk ratio, 95% CI)*	All women N = 619
Mean maternal age at booking, years (SD)		32.7 (5.1)	32.5 (4.7)	0.19 (-0.62–0.99)	32.6 (5.0)
Ethnic group N (%)	White European	246 (60.7)	162 (75.7)	Ref	408 (67.0)
	Black African/Caribbean	114 (28.1)	29 (13.6)	2.09 (1.44–3.01)	143 (23.1)
	Asian	37 (9.1)	14 (6.5)	1.89 (0.87–4.11)	51 (8.20
	Other	8 (2.0)	9 (4.2)	0.93 (0.49–1.75	17 (2.7)
Risk factor N (%)	Previous spontaneous preterm birth	177 (43.7)	–	–	–
	Previous late miscarriage	138 (34.0)	–	–	–
	Previous PPROM	111 (27.4)	–	–	–
	Previous cervical surgery	143 (35.3)	–	–	–
	Incidental finding of a short cervix < 25 mm	4 (0.99)	–	–	–
Smoking status N (%)	Never smoked	283 (69.9)	171 (79.9)	Ref	454 (73.3)
	Current smoker	22 (5.4)	6 (2.8)	2.13 (0.88–5.15)	28 (4.5)
	Ex-smoker (gave up in current pregnancy)	29 (7.2)	11 (5.1)	1.54 (0.79–3.00)	40 (6.5)
	Ex (gave up before current pregnancy	69 (17.0)	26 (12.1)	1.49 (0.98–2.25)	95 (15.3)
	Unknown	2 (0.5)	0 (0)		
Mean BMI at booking kg/m^2^ (SD)		26.6 (6.9)	24.7 (4.6)	1.90 (0.98–2.81)	25.9 (6.2)
Current or past history of domestic violence N (%)		19 (4.7)	1 (0.5)	10.31 (1.39–76.45)	20 (3.2)
Current or past history of recreational drug use N (%)		11 (2.7)	7 (3.3)	0.83 (0.33–2.12)	18 (2.9)
Pregnancy outcome N (%)	Term delivery > 37 weeks	310 (77.1)	200 (95.7)	0.81 (0.76–0.98)	510 (82.4)
	Iatrogenic preterm delivery < 37 weeks	17 (4.2)	4 (1.9)	2.21 (0.75–6.48)	21 (3.4)
	Spontaneous preterm delivery < 37 weeks	75 (18.7)	5 (2.4)	7.80 (3.20–18.98)	80 (13.1)
	Spontaneous preterm delivery < 34 weeks	40 (10.0)	1 (0.4)	20.80 (2.88–150.21)	41 (6.7)
	Late miscarriage	14 (3.5)	0 (0)	–	14 (2.3)
	Premature pre-labour rupture of membranes	42 (10.4)	5 (2.3)	4.37 (1.75–10.87)	47(7.6)
Cervical shortening < 25 mm < 24 weeks in current pregnancy N (%)		71 (17.5)	Not measured	–	–
Received prophylactic or emergency intervention to prevent preterm birth N (%)	Abdominal or vaginal cerclage	105 (25.9)	0 (0)	**	105 (17.0)
	Progesterone beyond 14 weeks’ gestation	26 (6.4)	0 (0)	**	26 (4.2)
	Arabin pessary	6 (1.5)	0 (0)	**	6 (1.0)

*Quantitative variables analysed by regression with robust standard errors. Categorical variables analysed with X^2^ test.

**Comparison test not performed due to risk of treatment bias.

**Table 2:** Demographics of the INSIGHT cohort study population by outcome (*preterm iatrogenic deliveries excluded n = 21).

Characteristic	Sub-category	sPTB < 37 N = 80	Term delivery N = 510	Comparison (difference/risk ratio, 95% CI)*
Mean maternal age at booking in years (SD)		32.7 (6.3)	32.6 (4.7)	0.08 (− 1.36 to 1.52)
Ethnic group N (%)	White European	37 (46.3)	354 (69.4)	Ref
	Black African/Caribbean	27 (33.8)	107 (21.0)	1.82 (1.30 to 2.53)
	Asian	10 (12.5)	21 (4.1)	3.80 (1.91 to 7.57)
	Other	16 (20.0)	49 (9.6)	1.90 (0.84 to 4.34)
Risk factor N (%)	Previous spontaneous preterm birth	36 (45.0)	131 (25.7)	1.75 (1.32 to 2.33)
	Previous late miscarriage	37 (46.3)	93 (18.2)	2.54 (1.88 to 3.42)
	Previous PPROM	20 (25.0)	84 (16.5)	1.52 (0.99 to 2.33)
	Previous cervical surgery	14 (17.5)	124 (24.3)	0.72 (0.44 to 1.19)
	Incidental finding of a short cervix < 25 mm	0 (0)	2 (0.4)	–
Smoking status N (%)	Never smoked	62 (77.5)	369 (72.4)	Ref
	Current smoker	3 (3.8)	24 (4.7)	0.76 (0.23 to 2.44)
	Ex-smoker (gave up in current pregnancy)	3 (3.8)	36 (7.1)	0.52 (0.16 to 1.64)
	Ex (gave up before current pregnancy	12 (15.0)	79 (15.6)	0.92 (0.53 to 1.60)
	Unknown	0 (0)	2	–
Mean BMI at booking in kg/m^2^ (SD)		26.7 (5.0)	25.74 (6.5)	0.98 (− 0.24 to 2.19)
Received prophylactic or emergency intervention to prevent preterm birth N (%)	Abdominal or vaginal cerclage	33 (41.3)	69 (13.5)	**–**
	Progesterone beyond 14 weeks of gestation	12 (15.0)	13 (2.5)	**–**
	Arabin pessary	4 (5.0)	3 (0.6)	**–**

*Quantitative variables analysed by regression with robust standard errors. Categorical variables analysed with X^2^ test.

As expected, the sPTB rate in the high-risk group was far greater than in the low-risk group (18.7% vs 2.4% < 37 weeks). For those high-risk women who developed a short cervix, the sPTB rate was 22/71 (31.0%) < 34 weeks, and 30/71 (42.3%) < 37 weeks of gestation. The median (quartiles) gestational age of detection of cervical shortening was 18^+3^ weeks (16^+3^ to 20^+2^ weeks).

### Gestational profile of host response peptides

#### Elafin

**Figure 1:**
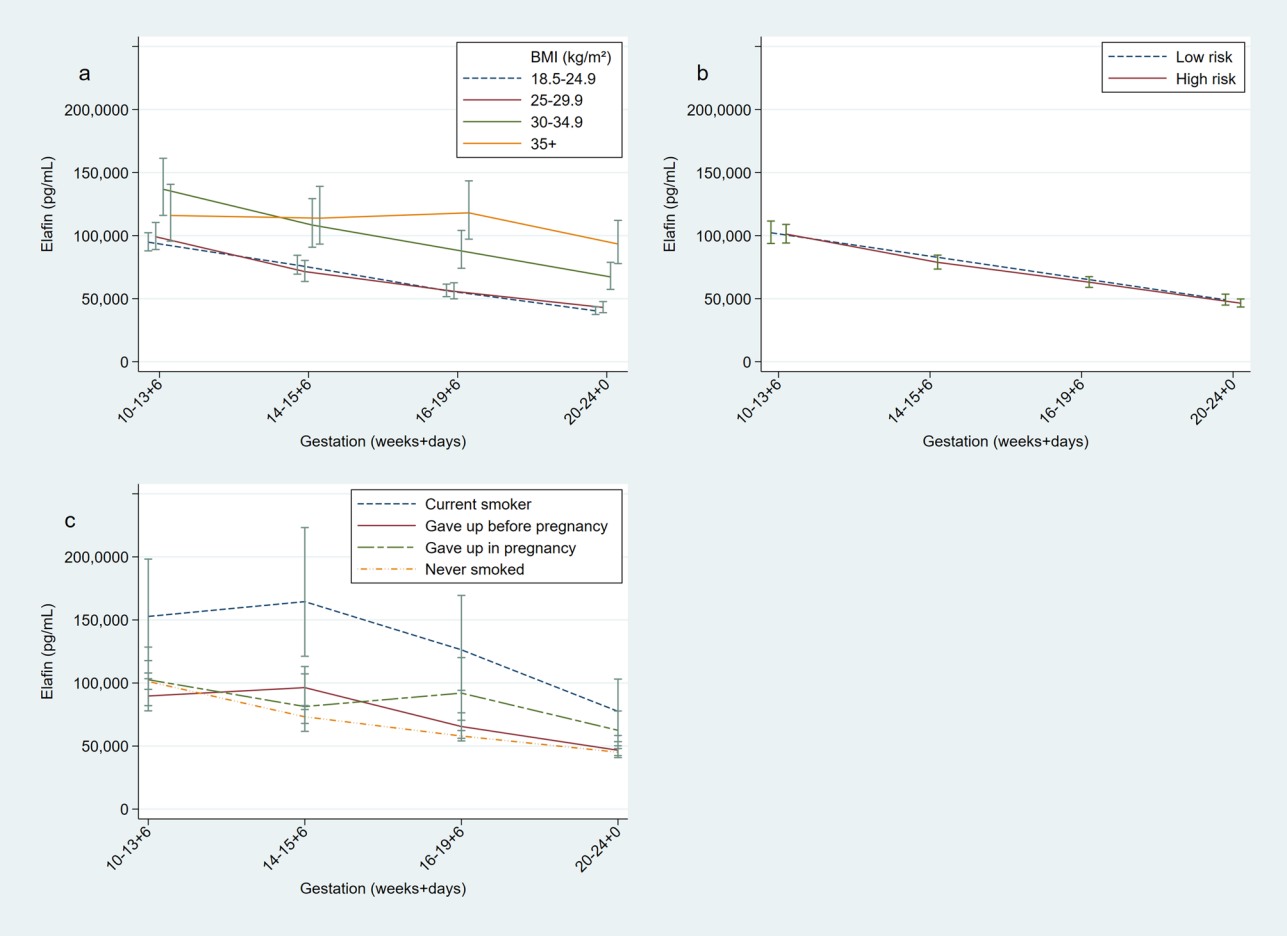
Longitudinal measurements of cervicovaginal concentrations of elafin measured by enzyme linked immunosorbent assay during pregnancy. Geometric means with standard error bars. (**A**) BMI < 24.9 (n = 303), BMI 25–29.9 (n = 163), BMI 30–34.9 (n = 68), BMI ≥ 35 (n = 48). (**B**) High risk women (n = 378), Low risk women (n = 207). (**C**) Current smoker (n = 28), ex-smoker, gave up before pregnancy (n = 95,), ex-smoker, gave up in pregnancy (n = 40), never smoked (n = 454). Figure prepared using StataCorp. 2015. Stata Statistical Software: Release 14. College Station, TX: StataCorp LP.

Across gestation there was a systematic decline in elafin CVF concentrations; mean values in the total cohort fell by approximately one half from 10 to 13^+6^ weeks of gestation to 20–24^+0^ weeks of gestation (101,624.2 ng/ml to 47,275.4 ng/ml, multiplier 0.47, 95% CI 0.42–0.52, p = 0.001). CVF elafin concentration reduced by 8.2% per week (0.92, 0.91 to 0.93, p < 0.001). CVF elafin concentration was found to be associated with maternal BMI (Fig. 1a). For every 1 kg/m^2^ rise in BMI, CVF elafin concentration rose by 1.7% (1.017, 1.003 to 1.031, p = 0.02). When considered categorically, a statistically significant rise in CVF elafin concentration was only associated with obese BMI compared to women with a BMI < 25 kg/m^2^; women with BMI of 30–35 kg/m^2^ had 52% (1.52, 1.15 to 2.00, p = 0.003) higher elafin concentrations and women with BMI > 35 kg/m^2^ had 65.0% higher elafin concentration (1.65, 1.21 to 2.24, p < 0.001). There was no significant difference in CVF elafin concentration according to risk status at enrolment (Fig. 1b).

Elafin concentration was strongly related to smoking status (Fig. 1c). Women who smoked in their current pregnancy, compared to women who reported having never smoked, had nearly double the CVF elafin concentration (1.97, 1.28 to 3.20, p = 0.002). There was no significant difference between women who gave up smoking before pregnancy, and women who had never smoked (1.05, 0.82 to 1.34, p = 0.681). There was no association between elafin concentration and maternal age. When BMI, current smoking status and elafin concentration were considered together using a multiple regression model, each remained independent predictors of elafin status (current smoking ratio 1.81, 1.17–2.81, p = 0.008, BMI 30–35 kg/m^2^ ratio 1.34, 1.01 to 1.79, p = 0.04).

#### Cathelicidin

CVF cathelicidin concentrations did not change significantly across gestation in high-risk women (multiplier per week 1.01, CI 0.99 to 1.03 p = 0.12). There was no association between cathelicidin concentrations and maternal BMI, or smoking status. Association was noted between maternal age and CVF cathelicidin concentration; for every increase in 10 years, CVF cathelicidin concentration reduced by 22% (95% CI 4% to 37%, p = 0.019).

#### Human neutrophil elastase

**Figure 2:**
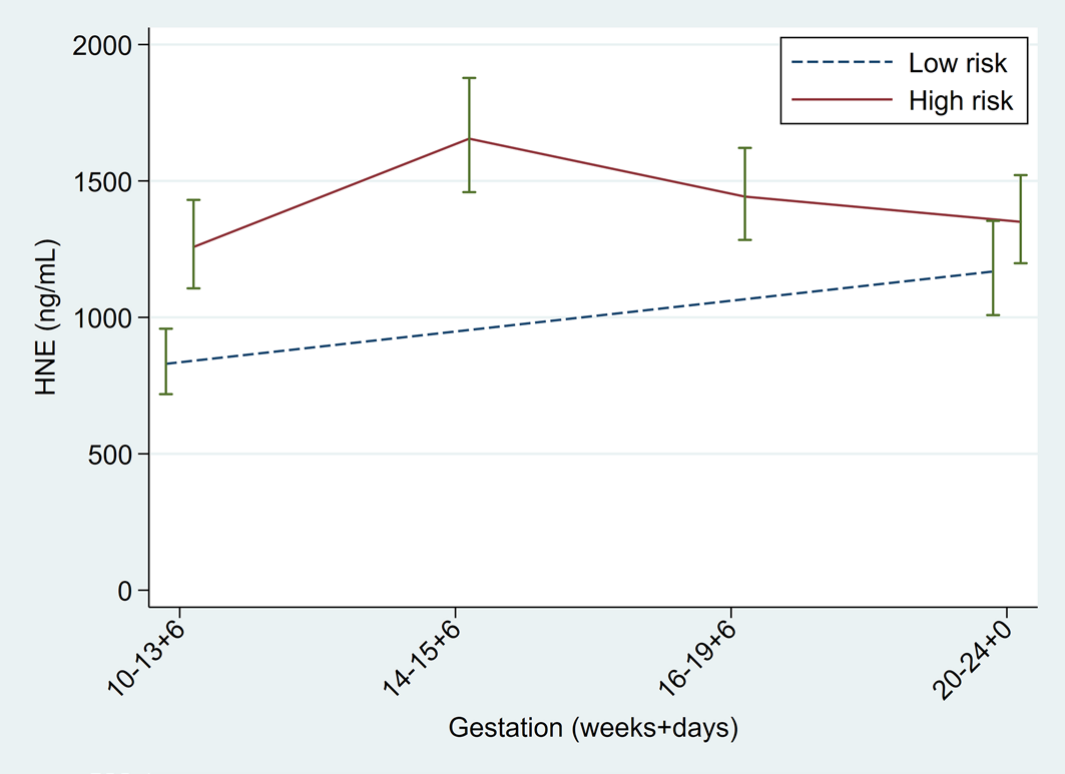
Longitudinal measurements of cervicovaginal human neutrophil elastase measured by enzyme linked immunosorbent assay during pregnancy. Geometric means with standard error bars. High-risk women (n = 358), Low-risk women (n = 205). Figure prepared using StataCorp. 2015. Stata Statistical Software: Release 14. College Station, TX: StataCorp LP.

CVF HNE concentration did not vary significantly across gestation (multiplier per week 1.01, 0.98 to 1.03, p = 0.323). Concentrations were 33% higher in high risk versus low risk women (Fig. 2, ratio 1.33, 1.01 to 1.76, p = 0.04), most marked at early gestations. There was no association between HNE concentration and maternal BMI, age or smoking status.

Table 3 describes the correlation between the CVF NAPs at each gestation. A positive correlation was detected between HNE and cathelicidin at each gestational sampling window. There was negative correlation between CVF elafin and cathelicidin concentration at early gestation (10 to 13^+6^ weeks’ gestation) and a positive correlation between elafin and HNE by mid-trimester (20 to 24^+0^ weeks’ gestation).

**Table 3:** Correlation between natural antimicrobial peptides (elafin, cathelicidin) and human neutrophil elastase in the whole cohort.

Gestation at visit (weeks^+days^)	Natural antimicrobial peptide/protein	Cathelicidin	HNE
10 to 13^+6^	Elafin	− 0.11 (1.43)	0.08 (1.3)
	Cathelicidin	–	**0.31 (< 0.001)**
14 to 15^+6^	Elafin	− **0.16 (0.02)**	0.02 (0.71)
	Cathelicidin	–	**0.47 (< 0.001)**
16 to 19^+6^	Elafin	− 0.11 (0.17)	0.05 (0.50)
	Cathelicidin	–	**0.47 (< 0.001)**
20 to 24^+0^	Elafin	− 0.09 (0.42)	**0.22 (< 0.001)**
	Cathelicidin	–	0.2 (0.07)

Data are Spearman’s correlation coefficient (p value).

Host response peptides, sPTB and adverse outcomes.

Values in bold are statistically significant at p < 0.05.

#### Elafin

In the whole cohort, mean CVF elafin concentrations, and when stratified by gestation at sampling, were not significantly different in women who delivered prematurely, and those who delivered at term, neither when analysed crudely (data not shown), nor after adjusting for maternal age, gestation at sampling, ethnicity, BMI, smoking and inter-plate pooled elafin concentration (Supplementary Table S1). Similarly, elafin concentrations were not associated with the pre-defined composite adverse maternal/fetal outcomes (Supplementary Table S1). Unadjusted elafin concentrations were not predictive (using ROC curve analysis) for sPTB before < 37 or < 34 weeks of gestation, nor maternal fetal/adverse outcomes (data not shown).

As seen in the whole cohort, when restricting the analysis to high-risk women only, adjusted CVF elafin concentrations were similar in women who delivered prematurely compared with term (data not shown) and those who developed cervical shortening prior to 24 weeks of gestation vs. those women who did not (Supplementary Table S1). Unadjusted elafin concentration did show weak prediction for cervical shortening when sampled in high-risk women in early pregnancy at 10^+0^ to 13^+6^ weeks (n = 232, ROC 0.58, 0.52 to 0.65) but not at any of the gestational sampling time-points.

After exclusion of samples taken after clinical intervention for cervical shortening had been initiated, unadjusted elafin concentrations measured at 10–13^+6^ weeks’ gestation were higher overall in women who delivered prior to 34 weeks’ gestation (n = 390, ratio 1.76, 1.00 to 3.10, p = 0.05) though this did not quite reach statistical significance, nor after adjustment (n = 387, 1.59, 0.93–2.80). Table 4 displays the ROC AUC for each outcome and gestation in high risk women in samples prior to intervention; high elafin was moderately predictive of subsequent sPTB < 34 weeks of gestation at 10 to 13^+6^ weeks of gestation (n = 202, AUC 0.63), 14 to 15^+6^ weeks of gestation (n = 211, AUC 0.61) and 20 to 23^+6^ weeks of gestation (n = 206, AUC 0.64). High elafin when measured at 10–13^+6^ weeks was predictive of cervical shortening (n = 203, AUC 0.60) and adverse fetal outcome (AUC 0.60, n = 207). In the whole cohort, CVF elafin was moderately predictive of sPTB < 34 weeks after exclusion of samples take post-intervention (Supplementary Table S2).

**Table 4:** Receiver operating characteristic area under the curve (95% confidence intervals) for prediction of outcome in high risk women, using CVF elafin concentration stratified by gestation at sampling, after exclusion of samples taken once prophylactic intervention had been initiated.

Outcome	Elafin receiver operating characteristic area under the curve (95% confidence interval)			
	Gestation category (weeks^+days^)			
	10–13^+6^	14–15^+6^	16–19^+6^	20–24^+0^
Cervical shortening < 25 mm	**0.60 (0.53–0.66)**	0.48 (0.42–0.55)	0.49 (0.43–0.56)	0.53 (0.46–0.60)
sPTB < 37 w	0.49 (0.42–0.56)	0.48 (0.41–0.55)	0.48 (0.41–0.55)	0.52 (0.44–0.59)
sPTB < 34 w	**0.63 (0.56–0.70)**	**0.61 (0.54–0.67)**	0.57 (0.50–0.63)	**0.64 (0.58–0.71)**
PPROM	0.54 (0.47–0.61)	0.52 (0.45–0.59)	0.49 (0.42–0.56)	**0.61 (0.53–0.67)**
Objective infection	0.49 (0.42–0.56)	0.49 (0.42–0.55)	0.50 (0.43–0.56)	0.53 (0.46–0.60)
Fetal adverse outcomes	**0.60 (0.53–0.67)**	0.50 (0.44–0.58)	0.48 (0.42–0.55)	0.52 (0.45–0.59)

Values in bold are statistically significant at p < 0.05.

#### Cathelicidin

**Figure 3:**
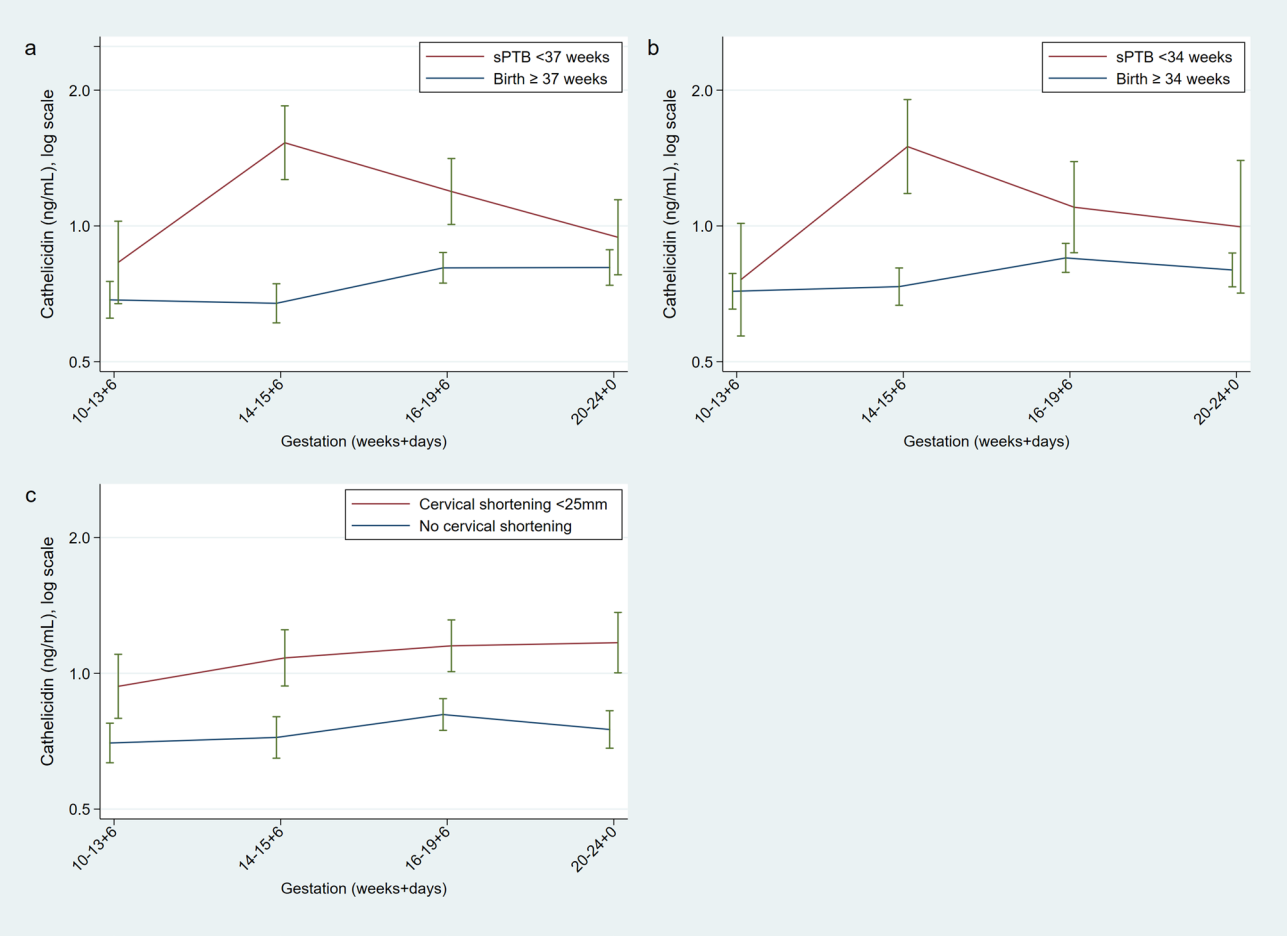
Longitudinal measurements of cervicovaginal cathelicidin concentrations in women at high risk of spontaneous preterm birth who deliver spontaneously prior to 34 weeks of gestation (n = 303 > 34 weeks and n = 36 < 34 weeks) (**A**) and prior to 37 weeks of gestation (n = 271 term, n = 68 preterm) (**B**) and women who subsequently develop a short cervix (n = 69 short, n = 254 not short) (**C**) Geometric means with SE bars. Figure prepared using StataCorp. 2015. Stata Statistical Software: Release 14. College Station, TX: StataCorp LP.

Overall, after adjustment, compared to women who delivered at term, CVF cathelicidin concentration was numerically 32% higher in high-risk women who delivered prematurely < 37 weeks (p = 0.04), and 17% higher in women who delivered < 34 weeks though this did not reach statistical significance (p = 0.38) (Table 5). Figure 3 illustrates the pattern of CVF cathelicidin concentration in sPTB cases and controls across gestation.

**Table 5:** Ratio (95% confidence intervals) of the logged mean CVF cathelicidin concentration in cases and controls (cervical shortening, preterm birth and maternal/fetal outcomes) overall, and stratified by gestation at sampling in high risk women.

Outcome	N (overall)	Overall	Ratio of cathelicidin concentration cases: controls*			
			Gestation category (weeks^+days^)			
			10–13^+6^	14–15^+6^	16–19^+6^	20–24
Cervical shortening < 25 mm	321	1.26 (0.96–1.65)	1.28 (0.82–1.99)	1.45 (0.89–2.38)	1.24 (0.91–1.70)	1.26 (0.85–1.87)
sPTB < 37 w	338	**1.32 (1.01–1.72)**	1.26(0.85–1.88)	**1.85 (1.18–2.92)**	1.30 (0.94–1.80)	1.14 (0.75–1.72)
sPTB < 34 w	338	1.17 (0.83–1.65)	1.10 (0.65–1.84)	1.88 (0.89–3.98)	1.19 (0.78–1.80)	1.09 (0.61–1.93)
PPROM	331	1.26 (0.90–1.78)	1.01 (0.61–1.65)	**1.94 (1.06–3.54)**	1.35 (0.89–2.03)	1.32 (0.76–2.28)
Objective infection	338	1.11 (0.82–1.51)	1.03 (0.64–1.67)	1.07(0.64–1.79)	1.29 (0.89–1.88)	1.28 (0.80–2.05)
Fetal adverse outcome	338	0.94 (0.71–1.26)	0.78 (0.50–1.23)	1.14 (0.71–1.83)	1.15 (0.82–1.62)	0.83 (0.53–1.29)

Values in bold are statistically significant at p < 0.05.

*Ratio adjusted for maternal age, gestation at sampling, ethnicity, BMI, smoking and inter-plate pooled elafin concentration.

Cathelicidin concentration in CVF samples taken at 14 to 15^+6^ weeks of gestation were 85% higher in women who delivered spontaneously prior to 37 weeks (p = 0.008) and nearly double in women who subsequently experienced PPROM (p = 0.03), compared to women who delivered at term (Table 5). When samples taken post-intervention were excluded, this association was strengthened; women delivering prior to 37 weeks of gestation had more than 2.5 times the CVF concentration of cathelicidin compared to controls at 14–15^+6^ weeks of gestation (n = 189, ratio 2.67, 1.59–4.47, p < 0.001), and more than double in women with PPROM (p = 0.03) (Supplementary Table S3). Overall, crude CVF cathelicidin concentrations were 35% higher in high-risk women who subsequently developed a short cervix by 24 weeks of gestation, compared to high-risk controls with normal cervical length (n = 323, ratio 1.35, CI 1.03 to 1.75, p = 0.03, Fig. 3c) although this statistical significance disappeared after adjustment (p = 0.09, Table 5).

At 14–15^+6^ weeks, CVF Cathelicidin was moderately predictive of sPTB < 37 weeks (ROC 0.67, 0.61 to 0.74), < 34 weeks (n = 229, ROC 0.63, 0.56 to 0.69) and PPROM (n = 225, ROC 0.64, 0.58 to 0.71) but not at other sampling timepoints, nor for adverse fetal or maternal outcomes. Cathelicidin concentrations were weakly predictive of developing a short cervix when sampled at 14–15^+6^ weeks (n = 232, AUC 0.58, 0.52 to 0.64), 16^+0^ to 19^+6^ weeks (n = 255, AUC 0.59, 0.53 to 0.65), and 20 to 24 weeks (n = 245, AUC 0.58, 0.52–0.64). Removal of samples taken after a woman received an intervention to prevent sPTB, prediction of sPTB by CVF cathelicidin was strengthened (Table 6). In particular, when sampled at 14–15^+6^ weeks, CVF cathelicidin predicted sPTB < 37 weeks with ROC AUC of 0.75 (n = 184).

**Table 6:** Receiver operating characteristic area under the curve (95% confidence intervals) for prediction of outcome in high risk women, using CVF cathelicidin concentration stratified by gestation at sampling, after exclusion of samples taken once prophylactic intervention had been initiated.

Outcome	Cathelicidin receiver operating characteristic area under the curve (95% confidence interval)			
	Gestation category (weeks^+days^)			
	10–13^+6^	14–15^+6^	16–19^+6^	20–24
Cervical shortening < 25 mm	**0.58 (0.51–0.66)**	0.58 (0.50–0.65)	**0.58 (0.51–0.65)**	0.49 (0.41–0.57)
sPTB < 37 w	**0.64 (0.56–0.71)**	**0.75 (0.68–0.81)**	**0.61 (0.53–0.68)**	0.58 (0.50–0.65)
sPTB < 34 w	**0.58 (0.51–0.66)**	**0.68 (0.61–0.75)**	0.57 (0.44–0.62)	0.53 (0.46–0.61)
PPROM	0.58 (0.50–0.65)	**0.67 (0.61–0.78)**	**0.65 (0.58–0.72)**	**0.66 (0.58–0.73)**
Objective infection	0.56 (0.49–0.63)	**0.62 (0.54–0.69)**	0.53 (0.46–0.60)	0.53 (0.45–0.60)
Fetal adverse outcomes	0.53 (0.45–0.60)	0.56 (0.49–0.63)	0.54 (0.47–0.61)	0.40 (0.33–0.48)

Values in bold are statistically significant at p < 0.05.

#### Human neutrophil elastase

Overall, CVF HNE concentration was not higher in women who delivered prematurely compared to women who delivered at term after adjustment for age, BMI, ethnicity, smoking, gestation at visit, and inter-assay plate variation (Supplementary Table S4), nor when stratified according to risk status at enrolment (data not shown).

**Figure 4:**
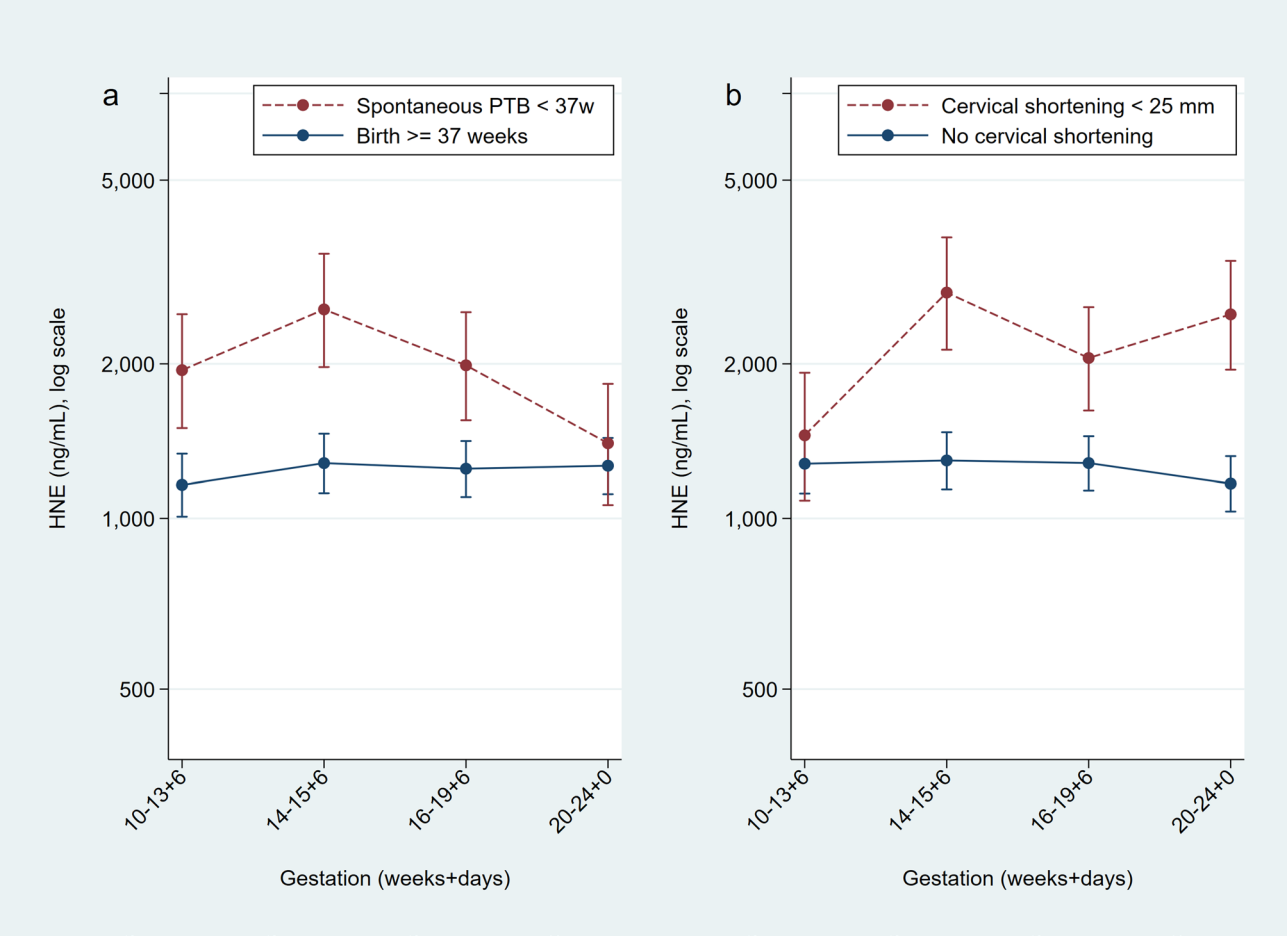
Longitudinal measurements of cervicovaginal HNE concentrations in women at high risk of spontaneous preterm birth who deliver spontaneously prior to 37 weeks of gestation (n = 281 term, n = 64 preterm) (**A**) and women who subsequently develop a short cervix (n = 62 short, n = 198 not short) (**B**). Geometric means and SE bars. Figure prepared using StataCorp. 2015. Stata Statistical Software: Release 14. College Station, TX: StataCorp LP.

When stratified by gestation, only crude concentrations of CVF HNE taken at 14–15^+6^ weeks of gestation were higher in women who delivered prematurely before 37 weeks (n = 246, ratio 2.25, 1.13–4.46, p = 0.02) and those who had PPROM (n = 253, ratio 2.95, 1.19–7.30, p = 0.02) compared to controls, but this did not reach statistical significance after adjustment (Supplementary Table S3). A similar trend was seen for association with adverse fetal outcomes (Supplementary Table S3). In this cohort, crude HNE concentrations were not predictive of sPTB using ROC analysis at any gestation. In high-risk women, however, both crude (ratio 2.43, 1.28–4.61, p = 0.007) and adjusted (Supplementary Table S3) CVF HNE concentrations were higher in women who later developed a short cervix compared to those who did not. Figure 4 shows the longitudinal expression of HNE in cases (sPTB) vs. controls. For this sub-group of high-risk women, crude HNE concentration at 14^+0^ and 15^+6^ weeks of gestation was modestly predictive of sPTB < 37 weeks (n = 236, AUC 0.63, 0.56 to 0.69), < 34 weeks (n = 241, AUC 0.61, 0.55–0.68), PPROM (n = 241, 0.65, 0.59 to 0.71), objective infection (n = 249, AUC 0.60, 0.53–0.66) and adverse fetal outcome (n = 249, AUC 0.60, 0.54–0.66). Similar results were seen when samples taken post intervention were excluded. In contrast, in later pregnancy (20–24 weeks of gestation), women who subsequently experienced sPTB < 34 weeks, and composite adverse neonatal outcome, had lower CVF HNE concentration than those women who did not (Supplementary Table S4).

## Discussion

This study described the longitudinal expression of host response peptides of the innate immune system and HNE in early to mid-trimester of pregnancy. Elafin expression was demonstrated to be highest in early pregnancy, most probably reflecting the suppression of elafin expression by oestrogen as pregnancy progresses^13^. Raised elafin concentrations were independently associated with smoking and obesity.

Constitutively expressed in the reproductive tract, elafin is further released from epithelial cells as a protective response initiated by inflammatory mediators, and/or is induced directly by microbial infection^14,15^. Our observations therefore most likely reflect the induction of elafin via sterile inflammation driven by increased adipose tissue and stimulated by components of cigarette smoke. Interestingly, neither HNE nor cathelicidin expression was influenced by these inflammatory states, nor did they change significantly across gestation.

We also sought to validate the association between CVF elafin and cathelicidin expression in early pregnancy with cervical shortening and subsequent sPTB, as well as undertake a preliminary investigation into the expression of HNE in a large cohort of low and high-risk pregnancies, in which 80 women spontaneously delivered preterm. We showed that CVF concentrations of cathelicidin and HNE (correlated with cathelicidin in this study) were higher in women who develop cervical shortening and who deliver prematurely, but that contrary to the hypotheses derived from our pilot study, high CVF elafin concentrations in early pregnancy were not associated with cervical shortening, nor sPTB in our large cohort of pregnant women some of whom received prophylactic treatment for a short cervix (unlike in the pilot study). However, removal of samples that were obtained after prophylactic treatment had been commenced in high-risk women revealed that high elafin in early pregnancy was modestly predictive of cervical shortening and subsequent sPTB < 34 weeks.

Like other NAPs, cathelicidin is expressed by neutrophils and epithelial cells and is co-released with HNE (as correlated in this study). Cathelicidin release can be induced by a range of inflammatory stimuli (e.g. Vitamin D, bacterial products, TNF alpha etc.), but this is complex and it can also act like a chemokine itself modulating immune cells^16^. In END1 cells, a human endocervical epithelial cell line, we found that cathelicidin was only induced by the active form of vitamin D (1,25-(OH)2) and calcipotriol and not by IL-1β or lipopolysaccharide^17^. Given that cervical ripening is associated with neutrophil influx and inflammation mediated by chemokines and cytokines^18^, and that we previously demonstrated strong association between CVF cathelicidin and cytokine concentration with cervical shortening^12^, the rise in CVF cathelicidin (and HNE) concentration prior to cervical shortening (median 18^+3^ weeks) was not unexpected. Cathelicidin has been shown to mediate the pro-inflammatory response in mouse models of term and sPTB with cathelicidin-deficient mice less susceptible to lipopolysaccharide induced sPTB^19^. Cathelicidin may have a responsive role to tissue damage around the time of cervical shortening, stimulating angiogenesis^20^, re-epithelialisation^21^, and promoting wound healing and immune cell infiltration. Cathelicidin and/or HNE may also be instrumental in the process of cervical shortening. HNE, for example, whilst targeting bacteria, can initiate proteolysis of collagen-IV and elastin^22^ found in cervical tissue, causing cervical damage and possibly shortening.

The more modest association between elafin and sPTB than suggested by the pilot study may be related to the complex pro and anti-inflammatory function that elafin plays as part of the innate immune system. High elafin could be protective against sPTB, limiting damage to host tissues by neutrophil activity by inhibiting neutrophil elastase and protease 3^23^, counteracting ascending infection and the associated cervical degradation and inflammation. However elafin may also cause tissue damage through its immunomodulatory actions, via neutrophil recruitment and release of further neutrophil elastase^24^, enhancing the inflammatory process. Furthermore, it is likely that elafin expression is influenced by the vaginal microbiota; Stock et al. found elafin to be lower in women with bacterial vaginosis (also associated with sPTB)^9^. Some bacterial species induce NAPs (e.g. *P. aeruginosa *^24^ whereas others (e.g. *E. coli, C. trachomatis*) suppress them^25,26^.

Associations between elafin and premature birth have been reported by other studies. Stalberg et al. demonstrated a twofold increase in elafin expression profiles in fetal membranes from six women with PPROM compared to six women who delivered at term^27^. In contrast, a case control study of 68 women of Japanese ethnicity reported, that cervical mRNA expression of elafin was increased in women in preterm labour who delivered prematurely compared with gestationally matched controls, and women in threatened preterm labour but who did not deliver prematurely^28^. However, in line with our study, a US cohort of 104 pregnant women^29^ (n = 26 sPTB) and UK cohort^30^ (n = 135 high risk women) failed to correlate CVF elafin concentration taken after 20 weeks of gestation albeit study sample processing was different.

As per UK clinical guidelines^31^, women in this study who had a previous sPTB or late miscarriage and who developed a short cervix were routinely offered prophylactic treatment such as cervical cerclage or vaginal progesterone, or an Arabin pessary. In addition, women may have received prophylactic intervention in early pregnancy. In contrast to our previous pilot study, we did not exclude women who received prophylactic intervention (the highest risk population), nor could we ethically withhold treatment. However, it was reasonable to stratify our analysis by risk status (the pilot study included only women with a previous history of sPTB and late miscarriage, in whom the pathology of subsequent sPTB may be different from a low-risk cohort) and also to exclude samples obtained after prophylactic intervention had been initiated (treatment may affect CVF protein expression, as well as cervical length and/or pregnancy outcome). By excluding samples taken once intervention had been started (cerclage, progesterone pessaries, Arabin pessary), we were able to rule out an intervention-mediated biomarker shift, but it is still possible that women destined to deliver preterm (with corresponding biomarker expression), may have had their delivery delayed by intervention, weakening the apparent predictive capacity of any NAP biomarkers.

The more modest relationship with elafin and pregnancy outcome in this study, compared to the pilot study is a reminder of the need to validate small studies in large powered cohorts. It may be that the pilot study was subject to Type 1 error by virtue of numbers (n = 74). Furthermore, whilst sample acquisition, preparation and protein measurement was performed using the same methodology, allowance for the dilutional effect of sample buffer on measured elafin protein concentration was not considered in the pilot; control samples were diluted to 1:10, and cases to 1:50 or 1:100 to ensure positioning on the standard curve. This may have led to some overestimation of true protein concentration in cases whereby dilution of samples has encouraged protein release from the cell matrix, or underestimation of control sample concentration. By correcting for the dilution effect here, we have presented a more accurate representation of protein concentration within the limitation of ELISA methodology.

## Conclusions

In conclusion, we have demonstrated evidence supporting the contribution of antimicrobial peptides to the mechanisms leading to some sPTB. Further investigation of this complex innate immune response is warranted to further understand their role in mediating risk of sPTB, and the potential to harness this information to develop more sophisticated prediction and prevention techniques than currently clinically utilised. In particular, the relationship between elafin and cathelicidin and specific sPTB phenotype subgroups, as well as correlation with the vaginal environment (i.e. the vaginal microbiota and cytokine expression), could provide additional insight and this is being explored in our cohort. Of the two CVF antimicrobial peptide/proteins measured here, mid-trimester cathelicidin has promising biomarker potential, and combination with other inflammatory markers, microbial colonisation and/or other NAPs may prove clinically useful in future.

## Methods

### Study population and design

A cohort of women with singleton pregnancies between 10^+0^ and 24^+0^ weeks of gestation considered to be at high-risk of sPTB were prospectively recruited from four tertiary UK high-risk antenatal clinics. High-risk was defined by at least one of; prior sPTB or late miscarriage between 16 and 37 weeks of gestation, previous destructive cervical surgery or incidental finding of a cervical length < 25 mm on transvaginal ultrasound scan. Low risk controls (singleton pregnancies between 10 and 24^+0^ weeks of gestation with no risk factors for sPTB, and otherwise well at the time of recruitment) were recruited from the routine antenatal or ultrasonography clinics at these centres. Exclusion criteria for both the high and low risk groups were multiple pregnancy, known major congenital fetal abnormality, rupture of membranes or current vaginal bleeding.

High risk women, at recruitment, were asked to consent to provide samples (at least once) between; 10–13^+6^ weeks, 14 to 15^+6^ weeks, 16 to 19^+6^ weeks and 20 to 24^+0^ weeks’ gestation, depending on the gestation they were recruited. Study participation did not affect choice of sPTB prophylaxis or treatment in the event of a short cervix (cerclage, progesterone or Arabin pessary). Clinical management decisions were made at the discretion of the attending clinician unless women opted to participate in a parallel randomised clinical trial (SuPPoRT)^32^. Low-risk women were seen, and vaginal swabs taken with consent on up to two occasions, once between 10 and 15^+6^ weeks, and once at 16–24^+0^ weeks of gestation. Visits usually coincided with dating and anomaly ultrasound scan visits.

For this study, which required at least n = 45 sPTBs for validation of previous findings (see power calculation below), samples were selected from all INSIGHT recruits for whom final visit data, samples and estimated due dates were on or before 05/03/2017. All high-risk women within this time frame were included if they had donated at least one sample. Women from the low risk control group were included for analysis if they had provided two longitudinal samples. Primary outcomes were development of a short cervix, and also sPTB birth prior to 34 and 37 completed weeks’ gestation. Secondary outcomes included premature pre-labour rupture of membranes, composite evidence of infection at delivery (any of maternal pyrexia during labour > 38°; raised c-reactive protein (CRP) > 10 or white blood cell count (WBC) > 20 during labour or within 24 h after delivery; clinical diagnosis of chorioamnionitis; positive maternal blood culture, midstream urine or high vaginal swab during delivery admission), and composite poor neonatal outcome (any of Apgar score < 5 at 1 or 5 min at delivery; admission to special care baby unit/neonatal intensive care unit (SCBU/NICU); intraventricular haemorrhage; ultrasound brain abnormality; respiratory distress syndrome; necrotising enterocolitis, NICU/oxygen at 28 days, hypoxic ischaemic encephalopathy; neonatal death; positive culture of infection in first 48 h).

Outcome data was obtained from case note review by trained study team members. Women were considered to have had a sPTB if labour was spontaneous in onset or there was premature rupture of membranes and delivered prior to 37 weeks of gestation (including late miscarriages), regardless of mode of delivery. Women with iatrogenic delivery (including those delivered due to maternal medical conditions and intrauterine fetal demise) were excluded from the analysis only if delivery occurred before the specific gestational outcome of interest (n = 21 < 37 weeks, n = 11 < 34 weeks).

### Cervical length assessment

Cervical length measurement by transvaginal ultrasound scan was performed by trained operators in accordance with standardised guidelines as per clinical protocols (at least once between 14 and 24 weeks’ gestation, usually at every clinical visit), with the woman in supine position with an empty bladder. A sagittal view of the cervix was obtained with the long axis view of the endocervical mucosa. Transfundal pressure was exerted to assess funnelling, and the total closed length in all women was measured 3 times with the shortest measurement recorded. For analysis purposes, the cervix was classified as ‘short’ if it measured less than 25 mm prior to 24^+0^ weeks’ gestation.

### Specimen collection and sample processing

At each visit a Dacron swab was used to obtain CVF from the posterior fornix via speculum examination for approximately 10 s to achieve saturation, and then inserted into 750 µl of standard phosphate-buffered saline solution containing protease inhibitors (Complete, Roche Diagnostics GmbH, Germany). This was immediately transported on ice to the laboratory. The swab was removed, placed in a clean tube, vortexed for 10 s and centrifuged (2,600×*g* for 10 min at 4 °C). Resultant fluid was collected and added to the fluid in the original tube. This was mixed and centrifuged for a further 10 min to remove cell debris. Cell-free supernatants were divided into aliquots (∼ 110 µl) and stored at − 80 °C until analysis^10^.

### ELISA for elafin, cathelicidin and HNE

Samples were thawed at room temperature, briefly vortexed and analysed by enzyme-linked immunosorbent assay [Trappin2/elafin, HK318; cathelicidin (LL-37), HK321; HNE, HK319-02, all from Hycult, Biotech Cambridge] in duplicate, according to manufacturer's instructions. Samples used for elafin measurement were diluted in sample buffer (1:20 and 1:100 for each sample) to ensure positioning within the standard curve, based on results obtained from a pilot study (CLIC). Samples used for HNE measurement were diluted in sample buffer 1:200. CVF samples for cathelicidin measurement were undiluted. A consistent ratio of samples from high and low risk women were included on each plate to minimise bias. A pooled CVF sample was created by combining a random set of 10 CVF samples from the pilot study and included on each plate to correct for inter-assay variability (< 15%). Final concentrations were calculated from the standard curves using logistic regression. For trappin2/elafin, the detection range of the ELISA was 0.9 to 10 ng/ml. For cathelicidin this was 0.1 to 200 ng/ml and for HNE, 5 to 5,000 ng/ml. Elafin was detectable in all but one of the CVF samples (range 3.999 to 1,062.059 ng/ml). Cathelicidin (range 0.001 to 12.6 ng/ml) and HNE (range 0.4 to 7,500 ng/ml) were detected in all the samples tested. Accepted coefficient of variability (CV) between sample duplicates was < 20%.

Of the 619 women enrolled, 1521 CVF samples from 586 women yielded usable elafin measurements (Table 7). Cathelicidin was measured in a subgroup of high-risk women (976 samples from 342 women) in whom there was sufficient CVF sample after the elafin analysis was complete. A total of 1,495 samples from 577 women were used for HNE.

**Table 7:** Number of valid cervicovaginal fluid samples provide by women at high and low risk of spontaneous preterm birth for each host response peptide collected at each gestational category.

Risk status	Host response peptide	Gestation at sampling (weeks^+days^)				Total
		Category 1 10–13^+6^	Category 2 14–15^+6^	Category 3 1619^+6^	Category 4 20–24^+0^	
High risk women	Elafin	241	265	313	297	1,116
	Cathelicidin	215	234	268	259	976
	HNE	233	251	317	288	1,089
Low risk women	Elafin	193	9	18	185	405
	HNE	194	10	18	184	406

These figures include all samples which may have been taken more than once in each gestational sampling window.

### Statistical analysis

Based on the pilot data (elafin as a predictive biomarker for sPTB) we calculated that 300 high-risk women would provide 40–50 cases of sPTB < 37 weeks of gestation. Assuming a threshold with 90% specificity, 45 cases would give 83% power to distinguish a relatively weak test (sensitivity 60%) from a relatively strong test (sensitivity 80%).

Analysis was performed using Stata (version 14.2, Stata Corp, College Station, Texas). Following distributional plots, log transformations were used as needed. Elafin showed censoring of low and high values in both the 1:20 and 1:100 diluted samples. In matched samples, elafin concentrations from 1:100 dilution were higher than from 1:20, and a multiplier of 0.535 was needed. Interval regression was used, with undetectable concentrations taken as between zero and the smallest concentration observed^33^. Values above the limit of accuracy (even at 1:100 dilution) were likewise taken as being above the upper limit of normal.

Differences between cases and controls were corrected for gestation and inter-assay plate variation. Random effects models allowed for repeated observations by participant. For elafin, where values were censored due to measurement limits, a random effects interval regression model was used. Spearman’s rank correlations (r_s_) show association between markers. Actual p-values are given (usually to 2 decimal places), except for very small values, shown as p < 0.001.

Prediction analysis used the first sample in each gestational age group: 10–13^+6^, 14–15^+6^, 16–19^+6^ and 20–24^+0^ weeks at time of sample. Missing measurements during each of these time-points were due to clinic non-attendance/consent not given for collection, enrolment after a gestational time period had expired, or invalid samples (e.g. vaginal bleeding at the time of swab, high CV duplicate values, or invalid ELISA plate reading and no repeat sample available). ROC curves with exact binomial confidence intervals^34^ were used for sPTB, maternal/fetal outcomes and cervical shortening. In a sensitivity analysis, samples taken after women received progesterone, cerclage or Arabin pessary (which may affect both inflammatory biomarker expression and/or pregnancy outcome) were excluded.

### Ethical approval and consent to participate

Biological samples for analysis were obtained from a sub-set of women recruited to the ongoing prospective observational study; INSIGHT: Biomarkers to predict premature birth. Approval from London City and East Research Ethics Committee was granted (13/LO/1393). Informed written consent was obtained from all participants.

## Supplementary information

Supplementary Information
