# Supplementary material for: Cervicovaginal natural antimicrobial expression in pregnancy and association with spontaneous preterm birth
Source: Sci Rep. 2020 Jul 21;10:12018. doi: 10.1038/s41598-020-68329-z (PMC7374562; doi:10.1038/s41598-020-68329-z)
Supplement: Supplementary file 1 — Supplementary Information [file 41598_2020_68329_MOESM1_ESM.docx]

**Cervicovaginal natural antimicrobial expression in pregnancy and association with spontaneous preterm birth**

Natasha L Hezelgrave*, Paul T Seed, Evonne C Chin-Smith, Alexandra E Ridout,

Andrew H Shennan, Rachel M Tribe

Dept. of Women and Children's Health

School of Life Course Sciences

King's College London

St Thomas' Hospital

London SE1 7EH

0207 188 3639

* Corresponding author

Supplementary Table 1. Ratio (95% confidence intervals) of the logged mean CVF elafin concentration in cases and controls overall, and stratified by gestation at sampling in the whole cohort (high risk and low risk)

| Outcome |  | Ratio of elafin concentration cases: controls** | | | | | |
| --- | --- | --- | --- | --- | --- | --- | --- |
|  |  |  | **Gestation category (weeks^+days^)** | | | | |
|  | **N (overall)** | **Overall** | | **10-13^+6^** | **14-15^+6^** | **16-19^+6^** | **20-24** |
| Cervical shortening <25 mm*** | 371 | 0.96(0.73-1.26) | | 1.08 (0.71-1.64) | 0.73 (0.50-1.06) | 0.78 (0.56-1.09) | 0.95 (0.67-1.35) |
| sPTB <37 w | 553 | 0.95 (0.74-1.22) | | 0.86 (0.61-1.22) | 1.10 (0.75-1.60) | 0.99 (0.70-1.38) | 1.09(0.77-1.54) |
| sPTB <34 w | 562 | 1.05 (0.75-1.48) | | 1.14 (0.69-1.88) | 1.32 (0.83-2.09) | 1.04(0.66-1.64) | 1.10 (0.66-1.84) |
| PPROM | 565 | 1.00 (0.73-1.37) | | 0.86 (0.57-1.31) | 1.28 (0.79-2.10) | 1.02 (0.65-1.61) | 1.10 (0.70-1.73) |
| Objective infection | 573 | 0.94 (0.73-1.23) | | 0.86 (0.60-1.24) | 0.88 (0.57-1.35) | 0.90(0.60-1.36) | 1.18(0.84-1.66) |
| Fetal adverse outcome | 573 | 0.92 (0.72-1.20) | | 0.94 (0.65-1.37) | 1.02 (0.69-1.51) | 0.96(0.67-1.37) | 0.85 (0.60-1.20) |

***High risk women only

**Ratio adjusted for maternal age, gestation at sampling, ethnicity, BMI, smoking and inter-plate pooled elafin concentration

Supplementary Table 2. Receiver Operating Characteristic Area under the Curve (95% confidence intervals) for prediction of outcome in all women (high risk ad low risk), using CVF elafin concentration stratified by gestation at sampling, after exclusion of samples taken once prophylactic intervention had been initiated

| Outcome | Elafin Receiver Operating Characteristic Area Under the Curve (95% Confidence interval) | | | | | |
| --- | --- | --- | --- | --- | --- | --- |
|  |  | | **Gestation category (weeks^+days^)** | | | |
|  |  | **10-13^+6^** | | **14-15^+6^** | **16-19^+6^** | **20-24** |
| sPTB <37 w |  | 0.50 (0.45-0.55) | | 0.48 (0.41-0.55) | 0.48 (0.42-0.54) | 0.51 (0.45-0.56) |
| sPTB <34 w |  | **0.64 (0.59-0.69)** | | **0.61 (0.54-0.67)** | 0.57 (0.50-0.63) | **0.62 (0.57-0.67)** |
| PPROM |  | 0.52 (0.47-0.57) | | 0.52 (0.44-0.58) | 0.49 (0.42-0.55) | 0.53 (0.48-0.58) |
| Objective infection |  | 0.49 (0.44-0.54) | | 0.48 (0.42-0.55) | 0.50 (0.44-0.56) | 0.53 (0.47-0.58) |
| Fetal adverse outcomes |  | 0.55 (0.50-0.60) | | 0.50 (0.43-0.57) | 0.48 (0.42-0.55) | 0.48 (0.43-0.53) |

Supplementary Table 3. Ratio (95% confidence intervals) of the logged mean CVF cathelicidin concentration in cases and controls (cervical shortening, preterm birth and maternal/fetal outcomes) overall, and stratified by gestation at sampling in the high-risk women after post intervention samples were excluded

| Outcome |  | Ratio of cathelicidin concentration cases: controls* | | | | | |
| --- | --- | --- | --- | --- | --- | --- | --- |
|  |  |  | **Gestation category (weeks^+days^)** | | | | |
|  | **N (overall)** | **Overall** | | **10-13^+6^** | **14-15^+6^** | **16-19^+6^** | **20-24** |
| Cervical shortening <25 mm | 280 | 1.28 (0.93-1.77) | | 1.36 (0.87-2.13) | 1.45 (0.89-2.38) | 1.23 (0.82-1.82) | 1.43(0.71-2.90) |
| sPTB <37 w | 299 | **1.70 (1.26-2.30)** | | 1.51 (0.99-2.30) | **2.67 (1.59-4.47)** | **1.57(1.04-2.38)** | 1.53 (0.86-2.74) |
| sPTB <34 w | 299 | 1.31 (0.85-2.02) | | 1.14 (0.64-2.09) | 1.88 (0.89-3.98) | 1.44 (0.77-2.67) | 1.35 (0.50-3.59) |
| PPROM | 294 | 1.42 (0.93-2.16) | | 1.15 (0.67-1.97) | **2.35 (1.09-5.09)** | 1.40 (0.76-2.60) | 1.87 (0.73-4.80) |
| Objective infection | 299 | 1.16 (0.82-1.64) | | 1.40 (0.84-2.35) | 1.11 (0.62-1.99) | 1.13 (0.72-1.77) | 1.27 (0.66-2.42) |
| Fetal adverse outcome | 299 | 0.93 (0.66-1.30) | | 0.91 (0.55-1.49) | 1.14 (0.65-2.02) | 1.11 (0.70-1.76) | 0.54 (0.28-1.05) |

*Ratio adjusted for maternal age, gestation at sampling, ethnicity, BMI, smoking and inter-plate pooled elafin concentration

Supplementary Table 4. Ratio (95% confidence intervals) of the logged mean CVF HNE concentration in cases and controls (cervical shortening, preterm birth and maternal/fetal outcomes) overall, and stratified by gestation at sampling in the whole cohort (high risk and low risk)

| Outcome |  | Ratio of HNE concentration cases: controls** | | | | | |
| --- | --- | --- | --- | --- | --- | --- | --- |
|  |  |  | **Gestation category (weeks^+days^)** | | | | |
|  | **N (overall)** | **Overall** | | **10-13^+6^** | **14-15^+6^** | **16-19^+6^** | **20-24** |
| Cervical shortening <25 mm* | 347 | 1.57 (1.00-2.46) | | 1.15 (0.60-2.22) | **2.95 (1.52-5.68)** | 1.50(0.82-2.76) | 1.42 (0.75-2.68) |
| sPTB <37 w | 552 | 1.27 (0.83-1.95) | | 1.55 (0.80-3.01) | 1.81 (0.88-3.72) | 1.17 (0.62-2.18) | 0.60 (0.31-1.15) |
| sPTB <34 w | 552 | 1.05 (0.59-1.89) | | 1.31(0.51-3.33) | 1.90 (0.79-4.56) | 0.62 (0.26-1.40) | **0.27 (0.11-0.66)** |
| PPROM | 545 | 1.46 (0.86-2.50) | | 1.45 (0.65-3.20) | 2.31 (0.92-5.79) | 1.19 (0.53-2.70) | 1.05 (0.43-2.55) |
| Objective infection | 552 | 0.90 (0.57-1.41) | | 0.65 (0.34-1.26) | 2.25 (1.0-5.05) | 1.02 (0.49-2.12) | 1.09 (0.52-2.26) |
| Fetal adverse outcome | 552 | 0.84(0.54-1.32) | | 0.65 (0.32-1.32) | 2.04(0.99-4.23) | 0.81(0.42-1.57) | **0.36 (0.18-0.72)** |

*High risk women only

**Ratio adjusted for maternal age, gestation at sampling, ethnicity, BMI, smoking and inter-plate pooled elafin concentration
